# Top-down generated micro- and nanoplastics reduce macrophage viability without eliciting a pro-inflammatory response

**DOI:** 10.1186/s43591-025-00138-5

**Published:** 2025-08-01

**Authors:** Annemijne E. T. van den Berg, Kas J. Adriaans, Luke A. Parker, Elena M. Höppener, Hanna M. Dusza, Juliette Legler, Raymond H.H. Pieters

**Affiliations:** 1 Institute for Risk Assessment Sciences, Utrecht University, Yalelaan 104-106, 3584 CM Utrecht, The Netherlands; 2 TNO Environmental Modelling, Sensing and Analysis, Utrecht, The Netherlands

**Keywords:** Microplastics, Nanoplastics, Immune system, Toxicology, Macrophages, Secondary microplastics

## Abstract

The presence of micro- and nanoplastic particles (MNPs) in our environment, food and drinking water has raised public concern due to inevitable human exposure. MNPs can be intentionally added to products or formed from plastics through fragmentation in the environment. Macrophages may become activated upon encountering MNPs, potentially triggering inflammation. However, this process, particularly in response to fragmented MNPs, remains poorly understood. This study aims to investigate whether fragmented MNPs have cytotoxic and pro-inflammatory effects on human macrophages. We examined the immunotoxic effects of mechanically degraded secondary polyvinylchloride, polypropylene and polyamide particles (PVC, PP; < 1 μm and 1–5 μm, PA6.6; 1–5 µm), in addition to primary polystyrene beads (PS; 0.05, 0.2 and 1 μm) and titanium dioxide particles (TiO_2_; < 0.1 μm) on human THP-1 macrophages. After up to 24 h of exposure to 1, 10 and 100 μg/ml, uptake was determined through flow cytometry and confocal microscopy, and effects on macrophages were measured by assessing lysosomal activity, mitochondrial activity, lactate dehydrogenase leakage, NF-κB activity and cytokine secretion. PS particles were taken up by macrophages in a concentration-, time-, and size-dependent manner based on particle mass. Additionally, MNPs increased lysosomal activity, suggesting potential accumulation of the particles. Fragmented MNPs induced a decrease in mitochondrial activity and an increase in LDH leakage depending on concentration, specifying their cytotoxic potential. However, at these levels, they did not significantly induce NF-κB activity and cytokine production (IL-6, IL-1β, TNF-α). Our findings suggest a lack of a direct pro-inflammatory response by macrophages to fragmented MNPs of various polymer types. However, higher exposure concentrations induced cytotoxicity, which may indirectly influence immune system functioning. This work emphasizes the importance of studying environmentally relevant MNPs to provide deeper insights into potential health impact of physico-chemically altered MNPs.

**Supplementary Information:**

The online version contains supplementary material available at 10.1186/s43591-025-00138-5.

## Introduction

Micro- and nanoplastic (MNP) research is rapidly gaining momentum, driven by growing concerns regarding their potential impact on human health. Diverse reports have identified MNPs in daily life items such as bottled water, plastic food packaging, clothing, and personal care products, indicating that we are constantly exposed to MNPs [1]. MNPs are characterized as solid synthetic polymers that are less than 5 mm (microplastics) or less than 1000 nm (nanoplastics) in size and can exist in various shapes, including fragments and fibres [1–3]. They can be intentionally added to products (primary MNPs) or formed in the environment from larger plastics through fragmentation or degradation (secondary MNPs) [2]. In this study, we focus on mechanically fragmented MNPs, which represent a major and environmentally relevant source of plastic pollution [2]. Frequently detected polymer types include polyethylene (PE), polypropylene (PP), polystyrene (PS), polyvinyl chloride (PVC), polyethylene terephthalate (PET), and polyamides (PA) [4, 5].

Quantification of human MNP intake is challenging due to methodological limitations, and current intake estimations might be an underestimation as most methods generally exclude or are incapable of capturing the smaller size fractions of MNPs (< 100 nm) [6, 7]. Recently, a study by Leslie et al. discovered and quantified polymers in human blood, confirming human systemic exposure [8]. In addition, various polymer types have been found in tissues such as arteries, carotid artery plaque and the brain, although uncertainties remain regarding the correctness of this data [9–11]. The increasing awareness of exposure to MNPs has resulted in a growing interest in studying the potential adverse effects of MNPs on human health.

As the immune system plays a critical and systemic role in maintaining overall human health, alterations can have serious health consequences and therefore, it is important to investigate the immunotoxic effects of MNPs. An essential type of immune cell known to be responsive to particulate matter is the macrophage. They are recognized for their ability to phagocytose substances and activate other immune cells by releasing inflammatory cytokines such as IL-1β, IL-6 and TNF-α [12, 13]. Previous studies, including our own, have demonstrated that the uptake of PS particles by macrophages and other phagocytosing cells is size-dependent [14–17]. Particles larger than 0.5 µm can trigger active phagocytosis through surface receptor binding [18]. In contrast, particles smaller than 0.5 µm can be internalized through passive non-phagocytic pathways, resulting in particle accumulation within the cells that may persist until critical concentrations are reached, in particular if no elimination occurs [19]. In addition to assessing uptake, researchers have investigated the potential toxicity of PS on macrophages. Observed effects, if any, tend to manifest only at high concentrations [14, 20, 21].

Currently, research concerning the effects of various polymer types on the immune system remains limited and often involves only larger particle sizes [22, 23]. Numerous researchers investigating the toxicological effects of MNPs use the easy-to-obtain, commercially available primary PS beads as they are relatively simple to synthesize, size-specific, low in cost and easily brought in suspension [24, 25]. However, they cannot directly be compared to secondary MNPs, which are far more heterogeneous in size, shape and surface chemistry. Previously, we demonstrated that primary PS particles, when environmentally weathered, can phenotypically and functionally activate human monocyte-derived dendritic cells (MoDC), suggesting that weathering might affect the immunomodulatory capacity of the particles [17]. This suggests that secondary MNPs of different polymer types, shapes and sizes might induce activation of antigen-presenting cells such as macrophages, due to their unique characteristics [26].

Here, we tested in-house produced mechanically fragmented PVC, PP and PA6.6 particles and evaluated their impact on differentiated THP-1 macrophages. The MNP test materials are made up of distinct size fractions (< 1 or 1–5 µm) and meet the following three conditions important for their consideration as environmentally relevant test materials [27]: 1) they exhibit fragment or fibre particle morphology; 2) they demonstrate the intended size fractionation across a wide size range; and 3) their surface is unmodified by surfactants. The particles were thoroughly characterized and tested for unintended interference with the in vitro assays used in this study.

We investigated the uptake of different concentrations of fluorescent PS particles over time in THP-1 macrophages to understand the uptake kinetics of MNPs since reliable labelled fragmented MNPs are not yet available. In addition, cytotoxic and pro-inflammatory effects of fragmented MNPs were determined using different endpoints, including mitochondrial activity, membrane integrity, lysosomal activity, NF-κB activation and cytokine release. This allowed us to shed light on the immunomodulatory effects of diverse environmentally relevant polymer types, thus contributing to the assessment of human health hazards associated with MNP exposure.

## Materials and methods

### Cell culture

The human THP-1 cell line (American Type Culture Collection [ATCC]) was used to study macrophage activity. For NF-κB activity assays, a THP-1 Blue variant, which is a THP-1 cell stably transfected with an NF-κB reporter construct, was employed. The reporter system, based on secreted embryonic alkaline phosphatase (SEAP), allows for direct quantification of NF-κB pathway activation upon stimulation. THP-1 cells were cultured in DMEM/F-12 GlutaMAX (Gibco, 31,331–093) supplemented with 9% heat-inactivated Fetal Bovine Serum (Gibco, 10,500,064), 1% Penicillin/Streptomycin (Gibco, 15,140–122) and 1% Sodium Pyruvate (Gibco, 11,360–039). Cells were cultivated in T75 flasks at 37 °C and 5% CO_2_ and passaged twice a week by transferring 2 ml of homogenous cell suspension to new T75 flasks resulting in a cell suspension of 200,000 cells/ml. For experiments, cells were seeded into 48-well culture plates (Greiner CELLSTAR, 677,180) at 200,000 cells in 400 µl, and differentiated by adding 5 ng/ml phorbol 12-myristate 13-acetate (PMA; Sigma-Aldrich, P1585) for 48 h. After this, cells were washed once and allowed to rest in fresh supplemented medium for 24 h before exposure, following the protocol by Park et al. [28]. This low-dose PMA protocol induces a state of differentiation that does not pre-polarize the cells, thus preserving their innate responsiveness to subsequent stimuli. Flow-cytometry results confirmed expression of the macrophage marker CD14 after the differentiation (Figure S1). Fig. S3 shows that 88.4% of PMA-treated THP-1 cells were CD14⁺ after a 24 h rest period, consistent with an M0-like macrophage phenotype. The stable CD14 expression in the absence of cytokine secretion indicates that the cells were differentiated without polarisation toward M1 or M2 macrophage states.

### Generation and characterization of MNPs

The PP and PVC particles used in this study were prepared and provided by TNO as described previously [27]. Briefly, using a multi-step process consisting of milling under cryogenic conditions, ball-milling in 1-propanol and fractionation via sedimentation and sieving, PVC and PP material was created in size fractions of < 1 and 1–5 µm in a standard dispersion of 20 mg/ml in 1-propanol. The obtained material underwent comprehensive characterization using various techniques, including static light scattering (SLS), scanning electron microscopy with energy-dispersive X-ray spectroscopy (SEM–EDX), thermogravimetric analysis, and X-ray fluorescence [27]. In order to enhance the density of PP, a 30-wt% nano-talc, commonly utilized as a mineral filler in commercial PP, was introduced during the extrusion process. The resulting PP/talc is able to sink, ensuring effective contact with the cells, while still maintaining environmental relevance [27]. The PA6.6 particles (1–5 µm) were provided by Wessling as a suspension in 1-propanol. PA pellets were added to H_2_O_2_ and heated to 80 °C under stirring for 3 h. The pellets were then recovered and dried at 70 °C. The dried PA was cryogenically milled using a 20 mm stainless steel grinding ball and liquid N_2_ for 27 min. The subsequent powder was recovered using particle-free water and sieved to achieve the desired size fraction. Data of the characterisation by SLS, SEM, Raman and ICP-MS can be found in the sample passports of the MOMENTUM particles [29]. In addition, TiO_2_ (NM-101) particles in 1-propanol were used to compare the effect induced by the MNPs to a standard nanoparticle with known effects. These particles originate from the Joint Research Centre (JRC) repository [30]. Non-functionalized Fluoresbrite Yellow Green and plain PS particles were purchased in three different sizes; 0.05, 0.2, and 1 µm (Polysciences, Inc.). The fluorescent PS particles are internally dyed with fluorescein.

Scanning electron microscopy (SEM) was used to confirm the size and shape of the MNPs. Samples were prepared for SEM analysis by filtering suspensions of the microplastic particles over 25 mm gold or nickel coated polycarbonate filters under reduced pressure with pore sizes of 0.8 µm and 0.1 µm (TJ Environmental). After filtration, the filter was transferred onto an aluminum SEM-stub covered with carbon coated tape. The samples were coated with a thin conductive surface film of carbon using a carbon evaporator (Quorum Q150 T carbon evaporator) to make the microparticles electronically conductive. The SEM measurements were performed with a Tescan MAIA III GMH field emission scanning electron microscope equipped with a Bruker X-Flash 30 mm^2^ silicon drift energy-dispersive X-ray microanalysis detector. The SEM was operated using an acceleration voltage between 5–15 kV. SEM images were recorded using a secondary electron (SE) detector.

To determine the size distribution of the particles in 1-propanol, static Light Scattering (SLS) SLS was performed using a Shimadzu SALD 7500nano and a Horiba LA-960S2. Both systems were equipped with ~ 10 ml cuvettes capable of constant agitation via a paddle or magnetic stirrer, respectively. The dispersions were measured under constant movement at laboratory conditions (23 °C). The refractive indices (RI) used to calculate the particle size distributions from the scattering results were 1.55 for PVC, 1.60 for PP, 1.50 for PA6.6 and 1.58 for PS, respectively.

Additionally, we assessed the size distribution of the different particles in the supplemented medium (DMEM/F12 + 10% FBS, RI: 1.345) using a Mastersizer 3000 (Malvern Panalytical). Particles were incubated in medium for 2 h at a concentration of 100 µg/ml concentration. After incubation, a measuring cell was filled with ~ 5 ml of supplemented medium and 1 ml of the sample was added. Constant stirring was maintained using a magnetic stirrer at 600–800 rpm. The RI used for particle size distribution calculations were 1.593 for PVC, 1.490 for PP, 1.530 for PA6.6 and 1.588 for PS. Measurements were performed in non-spherical particle mode for PVC, PP and PA6.6 and in spherical particle mode for PS, with three measurements taken per sample. Data processing was performed using a general-purpose analysis model integrated within the Mastersizer software.

### Cell treatment & dosimetry

Prior to the experiments, stock suspensions of the particles were diluted in supplemented medium to obtain the desired concentrations. Although several recent studies provide estimates of MNP intake and have detected MNPs in several human tissues, significant uncertainties remain regarding the actual internalized dose in humans. This is due to the complex translation of external exposure into internal dose [6] and the technical limitations of current analytical techniques [11]. Therefore, the administered particle concentrations used in this study were 1, 10, and 100 µg/ml. These concentrations were selected to capture potentially environmentally relevant low exposures while defining the thresholds for adverse cellular responses, thereby providing a comprehensive dose–response profile for hazard identification. In addition to the particles, treatment of THP-1 cells with 10 ng/ml lipopolysaccharide (LPS; Sigma-Aldrich, Type O111:B4 from Escherichia coli) was used as a positive control to confirm pro-inflammatory activation. Based on uptake kinetics assessed for PS it was chosen to expose the cells for 24 h to measure cytotoxic and immunotoxic effects.

To make the exposure as controlled as possible, frequent vortexing was applied to the suspensions before dilution and exposure. In addition, the concentration of particles delivered to the cells was predicted using the Distorted Grid (DG) model. The DG model integrates sedimentation, diffusion, dissolution, adsorption, and polydispersity, making it a robust tool for investigating diverse MNPs and their complex interactions in biological media. A detailed description of the methods used are mentioned in the Report on in vitro dosimetry model and its applicability for the MOMENTUM MNPs (Version v1) [31]. Results show that the larger fractions (1–5 µm) sedimented faster than the smaller fractions (< 1 µm) and that the higher-density polymer types settled more rapidly than those with lower density. In short, PP < 1 µm and PP/talc 1–5 µm settle slowly, delivering less than 50% of the nominal dose to the cells within 24 h, while PA6.6 1–5 µm, with a Tmax of about 38.5 h, provide moderate exposure. In contrast, PVC < 1 µm and PVC 1–5 µm settle rapidly (Tmax of ~ 10.5 h and ~ 8 h, respectively), ensuring nearly complete cell exposure to the administered dose within 24 h. The exact predicted sedimentation metrics are mentioned in the report [31]. Table 1 shows the three nominal concentrations used in this study as the delivered mass after 24 h, using the fraction deposited for a 3 mm column height. The delivered dose of TiO2 was not estimated. However, due to its high density (4,23 g/cm^3^), we assume that the delivered dose is similar to the administered dose.

**Table 1:** Distorted Grid model–predicted delivered mass after 24 h for each test particle and nominal concentration

Particle	Size (µm)	*f*D max (%)	Delivered at 1 µg/ml	Delivered at 10 µg/ml	Delivered at 100 µg/ml
PVC	< 1	95.6	0.96	9.56	95.6
PVC	1–5	96.8	0.97	9.68	96.8
PP/talc	< 1	42.3	0.42	4.23	42.3
PP/talc	1–5	45.5	0.46	4.55	45.5
PA 6.6	1–5	86.8	0.87	8.68	86.8

*fD max* Maximum fraction delivered

### Analysis of PS uptake kinetics by flowcytometry and confocal microscopy

To provide information on uptake kinetics and to confirm earlier studies, THP-1 macrophages were exposed to 0.05, 0.2 and 1 µm fluorescent PS particles for 15 min, 3 h and 24 h in a mass-based concentration ranging from 1–100 µg/ml. Only primary internally labelled PS particles could be used due to the lack of reliably labelled fragmented MNPs. After exposure, cells were washed three times with PBS to remove particles that were not internalized by the cells and then cells were collected by adding 100 µl 2 mM EDTA/PBS for 20 min on ice. Cells were resuspended in 100 µl FACS buffer (FB; PBS containing 1% bovine serum albumin [BSA] and 0.1% NaN_3_). The amount of particle uptake was analyzed using a BD Accuri™ C6 flow cytometer and BD sampler software (BD Biosciences). The macrophages were gated using the forward and sideward scatter. Within this cell population, the percentage of positive cells, i.e. the percentage of cells that took up one or more PS particles, was determined by measuring the fluorescence per cell. Mean Fluorescent Intensity could not be used as a parameter since the data distribution was bi- and multimodal.

To confirm that the PS particles were taken up by the cells and not adhered to the surface, cells exposed to fluorescent PS particles were visualized using confocal microscopy. Briefly, 780,000 THP-1 cells were plated onto 18 mm coverslips and differentiated according to the earlier-mentioned protocol. Then, cells were exposed to 10 µg/ml of fluorescent PS particles (0.05, 0.2 and 1 µm) for 24 h. After the exposure, cells were fixed in paraformaldehyde (3.7%) for 15 min at room temperature and permeabilized with Triton X-100 (0.1% in PBS) for 15 min at room temperature. Then, cells were incubated in a staining solution containing Hoechst 33,342 (4.45 µM, Merck, 14,533), Alexa Fluor™ 568 Phalloidin (165 nM, Invitrogen, A12380) and 1% BSA, to stain nuclei and β-actin, respectively. In between each step, cells were washed twice in pre-warmed PBS. Finally, the coverslips were mounted onto slides using Prolong Diamond (Life Technologies, P36965) and left to harden overnight at room temperature. Cells were then imaged and z-stacks were created using a confocal microscope (LEICA TCS-SPE II). The images were processed using the ImageJ software.

### Brightfield microscopy for live cell morphology

The morphology of live cells was evaluated after 24 h of exposure to 100 µg/ml of the fragmented MNPs, TiO_2_ and LPS (10 ng/ml). At the 24 h time point, the plates were directly transferred to the Olympus CKX41 microscope with Leica DFC245C camera, equipped with a 40 × objective. No staining or fixation was performed to preserve the native morphology of the live cells and to visualize, where possible, the particle deposition and cell clustering. The images were processed using the LAS V4.31 software.

### Assessment of lysosomal activity

The lysosomal activity was measured in THP-1 cells after treatment by measuring the accumulation of Lysotracker red (DND-99, Invitrogen, L7528) in acidic cellular compartments as an indicator of total lysosome content. After exposure to the particles, the medium was removed and 200 µl of staining solution (100 nM Lysotracker Red and 2.5 µg/ml Hoechst) was added to each well and incubated for 1.5 h at 37 °C, 5% CO_2_. Cells were washed once with medium and fluorescence was measured using a Tecan Infinite M200 plate reader (Tecan Austria GmbH, 30,016,056) at excitation and emission wavelengths of 555 and 595 nm, respectively. Fluorescence values were normalized to the untreated cells. PS particles showed no signal in cell-free medium, excluding the potential of interference.

### Assessment of cytotoxicity via mitochondrial activity measurements and lactate dehydrogenase activity assay

In this study, we employed two cytotoxicity assays for hazard assessment: the AlamarBlue assay to assess mitochondrial activity and the LDH release assay to evaluate membrane integrity. We selected these surrogate markers of cell viability based on the rationale that a decrease in metabolic activity signals impaired mitochondrial function, while increased LDH release indicates cell membrane damage, both strongly associated with cell death. Together, these complementary assays offer a comprehensive view of the cytotoxic response. Since LPS served as a positive control for inflammation and not for cytotoxicity, we confirmed the sensitivity of the assays by testing a range of Triton X-100 concentrations, which demonstrated their reliability (data not shown). Effects of the MNPs on metabolic activity of the cells were measured using resazurin sodium salt (Sigma, 199,303). Following the exposure period, MNPs were washed away to avoid interaction of the MNPs with the reagent. Fresh medium containing 10% AlamarBlue reagent was added and cells were incubated for an additional 3 h at 37 °C, 5% CO_2_. Thereafter, the plate was centrifuged for 5 min at 200 g and 180 µl supernatant was pipetted into a black 96-well plate (Corning, 3916). Fluorescence was measured at excitation and emission wavelengths of 540 and 590 nm, respectively, using a Tecan plate reader. Fluorescence values were normalized to the untreated cells to obtain the relative metabolic activity. Additionally, as a control, milled talc particles were included in this assay. Particles were measured in medium without cells and did not show any interference with the fluorescence measurements.

After cell treatment, the LDH leakage assay was performed according to the manufacturer’s instructions (Sigma-Aldrich, MAK066). LDH activity was determined by measuring the absorbance at 450 nm at 5-min intervals until the value of the most active sample was greater than the value of the highest standard. Data was corrected for the background absorbance and the LDH activity was calculated by comparing the sample absorbance with the standard curve and reaction time. LDH activity values were normalized to the control conditions.

### Assessment of NF-κB signalling activation

To detect NF-κB induced transcriptional activation, we utilized a THP1-Blue cell line stably transfected with a secreted embryonic alkaline phosphatase (SEAP) construct. After treatment, a QuantiBlue assay was performed according to the manufacturer’s instructions to measure the SEAP enzyme activity correlating to the triggering of the NF-κB signalling pathway. Briefly, 20 µl of supernatant was mixed with 180 µl of QuantiBlue reagent (Invivogen, rep-qbs2) in a 96-well plate and incubated for 3 h at 37 °C, 5% CO_2_. The optical density was measured at a wavelength of 620 nm using a Tecan plate reader. The particles were also measured without cells to test for potential interference, but this was not the case.

### Cytokine quantification by Enzyme-Linked Immune-Sorbent assay (ELISA)

After exposure treatment, culture media from THP-1 cells were collected by centrifugation to remove the (larger) MNPs from the supernatant to avoid interference. Levels of cytokines IL-1β, IL-6 and TNF-α were quantified using ELISA kits (all from e-bioscience, 88–7261-88, 29–8069-65, 29–8321-65, respectively) according to the manufacturer’s instructions. The absorbance of each well was measured at 450 nm using a Tecan plate reader. To assess interference, cell-free particle samples were spiked with known amounts of each cytokine, and cytokine retrieval was measured after 24 h of incubation, confirming no interference.

### Statistical analysis

All results are presented as mean ± standard deviation (SD). Experiments were performed in triplicate, with three biological replicates per condition. Results are representative of one independent experiment unless otherwise stated. Statistical significance was determined by one-way or two-way analysis of variance (ANOVA) and *post-hoc* correction with Tukey’s multiple comparisons tests. Values of *p < 0.05 were considered as significant. All data were analyzed using Prism 8.3 software (GraphPad, San Diego, CA).

## Results

### Particle characterization

**Fig. 1:**
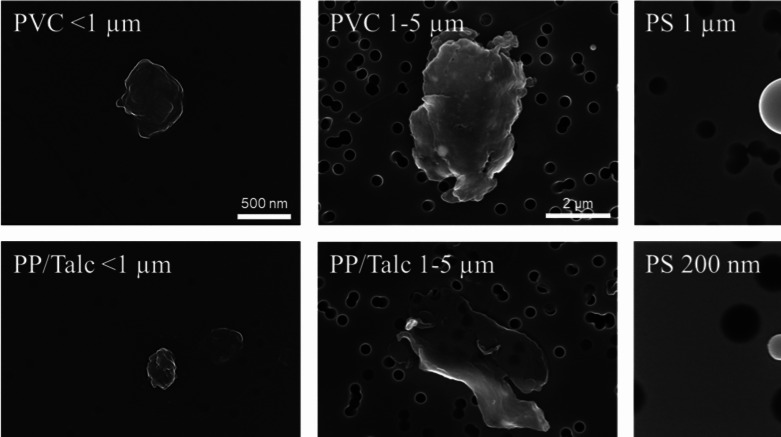
Characterization of micro- and nanoplastic particles by scanning electron microscopy (SEM). Representative Secondary Electron (SE) images of PVC and PP/talc (< 1 µm and 1–5 µm), PA6.6 (1–5 µm), TiO_2_ NM-101 (< 0.1 µm) and PS (50 nm, 200 nm and 1 µm)

Scanning electron microscopy (SEM) and Static Light Scattering (SLS) were performed to investigate the size and shape of the MNPs tested in the present study (Table 2 and Fig. 1). A clear difference in shape between primary and fragmented MNPs can be seen in Fig. 1, showing that the commercially available PS particles display a perfectly round spheric morphology compared to the irregularly shaped PVC, PA6.6 and PP/talc particles. The number average particle size (D_N_), represented as the median diameter in µm, was measured for both spherical and irregular particles in 1-propanol (stock solution) and in DMEM culture medium (Table 2). In DMEM, PVC particles smaller than 1 µm exhibited an increase in D_N_ from 0.29 µm to 2.77 µm, indicating agglomeration. Although to a lesser extent, PP/talc < 1 µm also demonstrated agglomeration in DMEM, with D_N_ increasing from 0.20 µm to 0.68 µm. Both PVC and PP/Talc in the 1–5 µm range did not show an increase in D_N_. However, PA6.6 particles (1–5 µm) exhibited a reduction in D_N_ from 3.63 µm in 1-propanol to 2.50 µm in DMEM. PS particles showed minimal change between the two media. Overall, PVC and PP/talc particles, particularly at smaller sizes, are more prone to agglomeration in DMEM compared to PA6.6 and PS particles. Figure S2 presents the number-based particle size distributions of the various samples in DMEM.

**Table 2:** Details of the tested MNPs. Using Static light Scattering (SLS), the median diameter on number basis (D_N_) was determined in 1-propanol and DMEM/F12 supplemented with 10% FBS

	Characteristics			SLS data	
Sample name	Material	Size (µm)	Shape	D_N_ (1-propanol, µm)	D_N_ (DMEM/F12, µm)
PS 0.05 µm	PS	0.05 µm	Spherical	0.07	0.06
PS 0.2 µm	PS	0.2 µm	Spherical	0.12	0.08
PS 1 µm	PS	1 µm	Spherical	1.14	0.91
PVC < 1 µm	PVC	< 1 µm	Irregular	0.29	2.77
PVC 1–5 µm	PVC	1–5 µm	Irregular	3.83	3.17
PP/talc < 1 µm	PP/talc	< 1 µm	Irregular	0.20	0.68
PP/talc 1–5 µm	PP/talc	1–5 µm	Irregular	3.63	3.18
PA6.6 1–5 µm	PA6.6	1–5 µm	Irregular	3.63	2.50

### Size-, dose-, and time-dependence of PS uptake by THP-1 cells

**Fig. 2:**
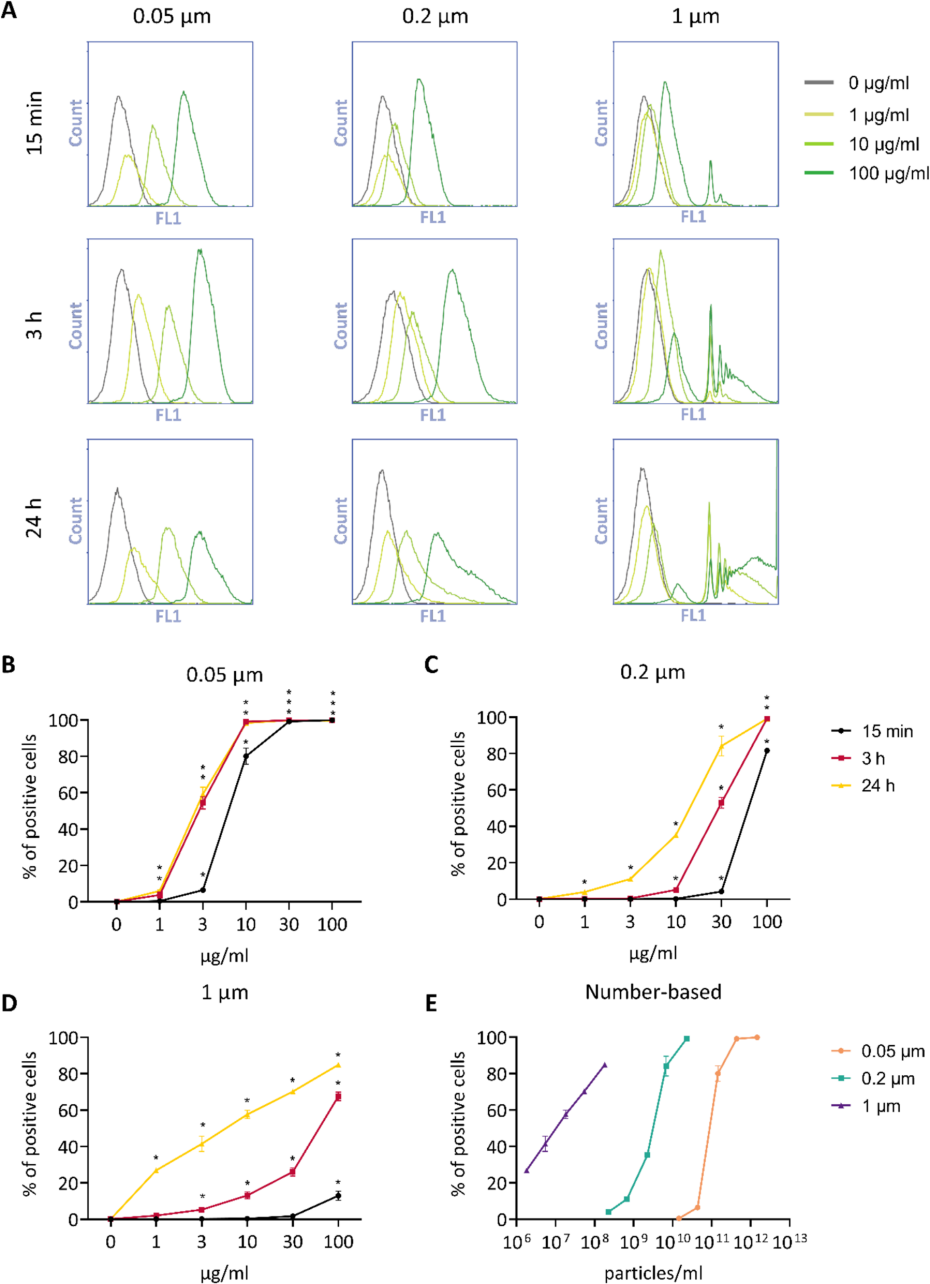
Uptake of YG fluorescent PS particles by THP-1 macrophages. A) Fluorescence intensity (FL1) histograms of THP-1 macrophages exposed for 15 min, 3 h and 24 h to 0, 1, 10 and 100 µg/ml PS particles (0.05, 0.2 and 1 µm). B-D) Mass-based uptake expressed by the percentage of positive cells. E) Particle-based uptake after 24 h of exposure. Data are presented as mean ± SD, *n* = 3. * *p* < 0.05 vs control

To assess the relevant incubation time and exposure levels, we tested the kinetics of uptake by the macrophages using labelled PS particles. Using flow cytometry, the fluorescence intensity (Fig. 2A) and the percentage of cells that had taken up MNPs (Fig. 2B-D) were measured. The 1 µm particles exhibit high fluorescence, allowing for individual detection upon uptake. This is visualized by the distinct Fluorescence Intensity (FL1) peaks shown in Fig. 2A. Analysis revealed that differentiated THP-1 macrophages engulfed all three sizes of polystyrene particles in a time-dependent manner. After 15 min of exposure to the highest concentration of PS particles, positive cells were observed for all sizes and by 24 h of exposure, more than 80% of cells had taken up one or more particles. Based on these results, 24 h exposure was used in further experiments. Figure 2E shows the uptake of PS particles by macrophages after 24 h exposure, considering the number of particles/ml rather than mass/ml. This figure highlights the difference in the number of particles to which the cells are exposed across different sizes.

**Fig. 3:**
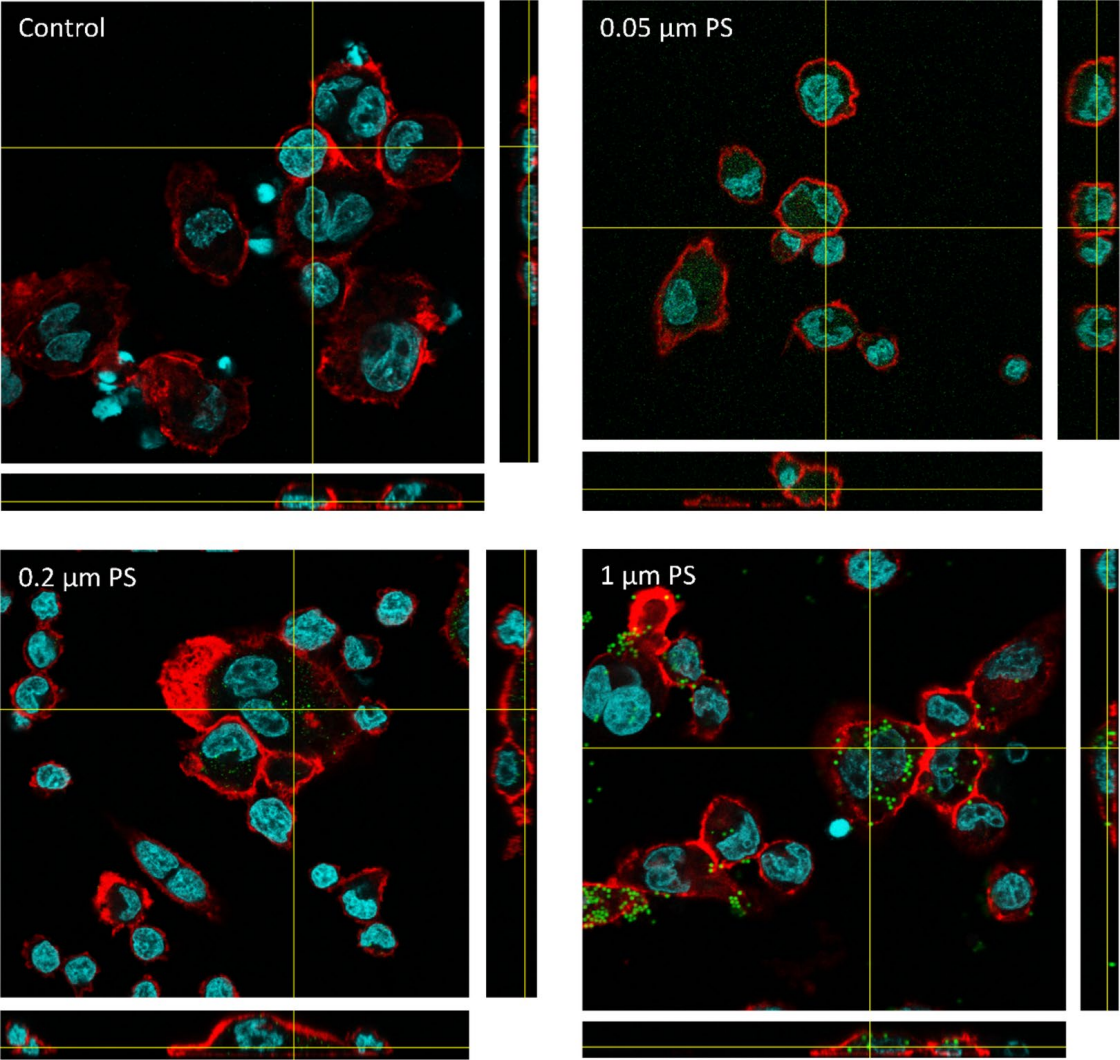
Confocal microscopy images of THP-1 macrophages exposed for 24 h to medium, 0.05, 0.2 and 1 µm YG fluorescent PS particles (10 µg/ml). Particles are shown in green, nuclei in cyan and actin in red. A colour-blind-safe image can be found in the supplements (Figure S3)

Confocal microscopy (Fig. 3) analysis further confirmed the uptake of PS by THP-1 macrophages. The different sizes of PS particles were observed to be localized inside the cells as visualized in the orthogonal views, suggesting that they were internalized through phagocytosis or endocytosis.

### THP-1 morphology

**Fig. 4:**
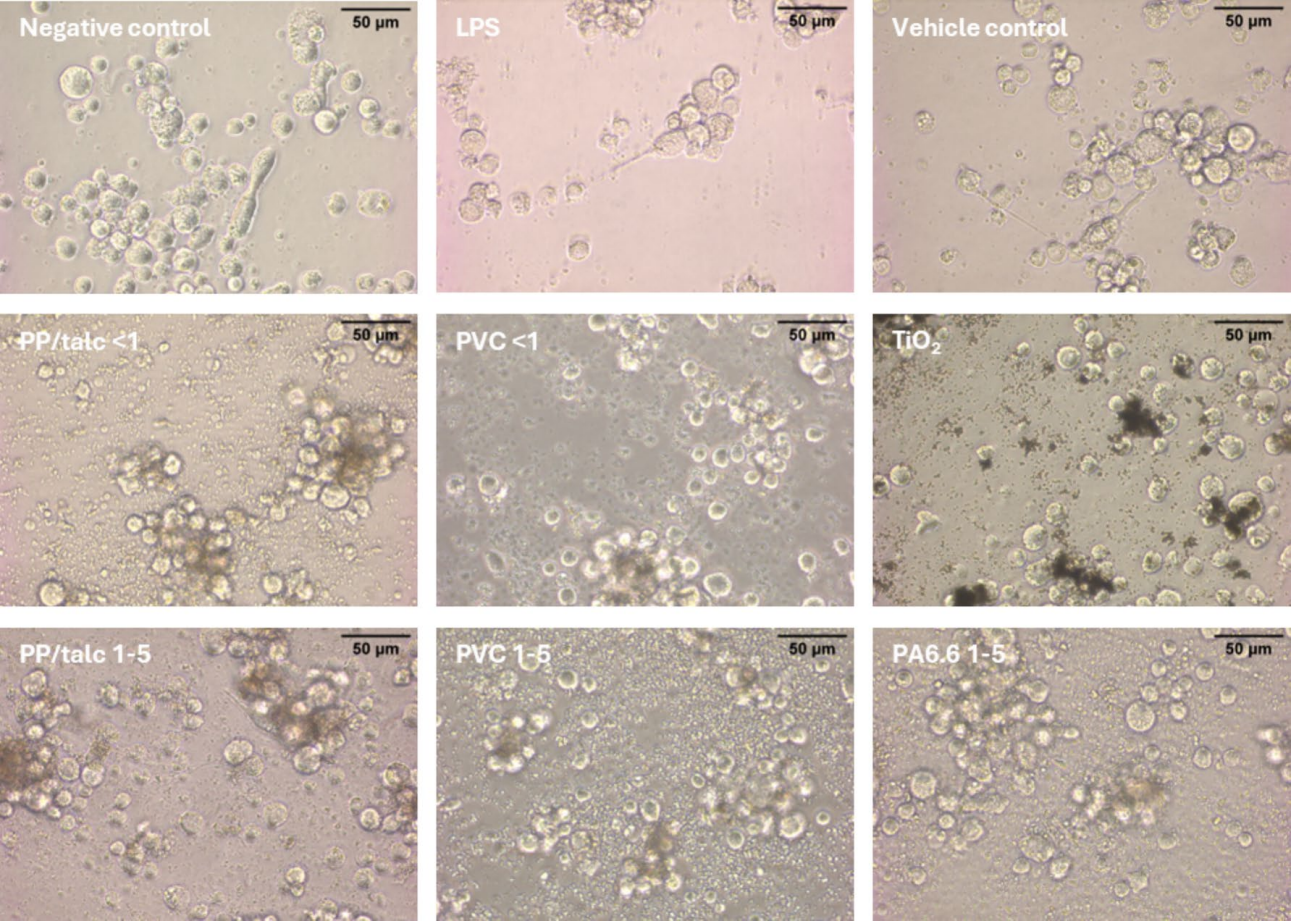
Representative brightfield micrographs of THP-1 macrophages after 24 h exposure to negative and vehicle controls, LPS (10 ng/ml), various microplastics (PA, PP, PVC), and TiO₂ (100 ug/ml)

Microscopic evaluation of PMA-differentiated THP-1 macrophages showed that both the negative (untreated) and vehicle (1-propanol) controls maintained the typical rounded or oval morphology of the cells present in small clusters and with minimal background granularity. LPS (10 ng/ml) exposure induced mild activation, with occasional cell elongation and protrusions, though most cells remained rounded. The TiO₂-exposed cells, serving as a particle control, exhibited distinct large, dark aggregates in the medium while retaining overall cell integrity (Fig. 4).

Following 24 h exposures to microplastics, PA6.6 (1–5 µm), PP/talc (< 1 µm or 1–5 µm), and PVC (< 1 µm or 1–5 µm), the THP-1-derived macrophages largely retained their round or oval shape. However, within the culture medium we observed granules or specks, consistent with the presence of suspended or aggregated particles. In many instances, the cells appeared closely associated with the microplastics, as indicated by localized darker or denser regions within cell clusters. Despite these interactions, there was no evidence of widespread cell lysis or blebbing.

### Activation of lysosomes by MNPs

**Fig. 5:**
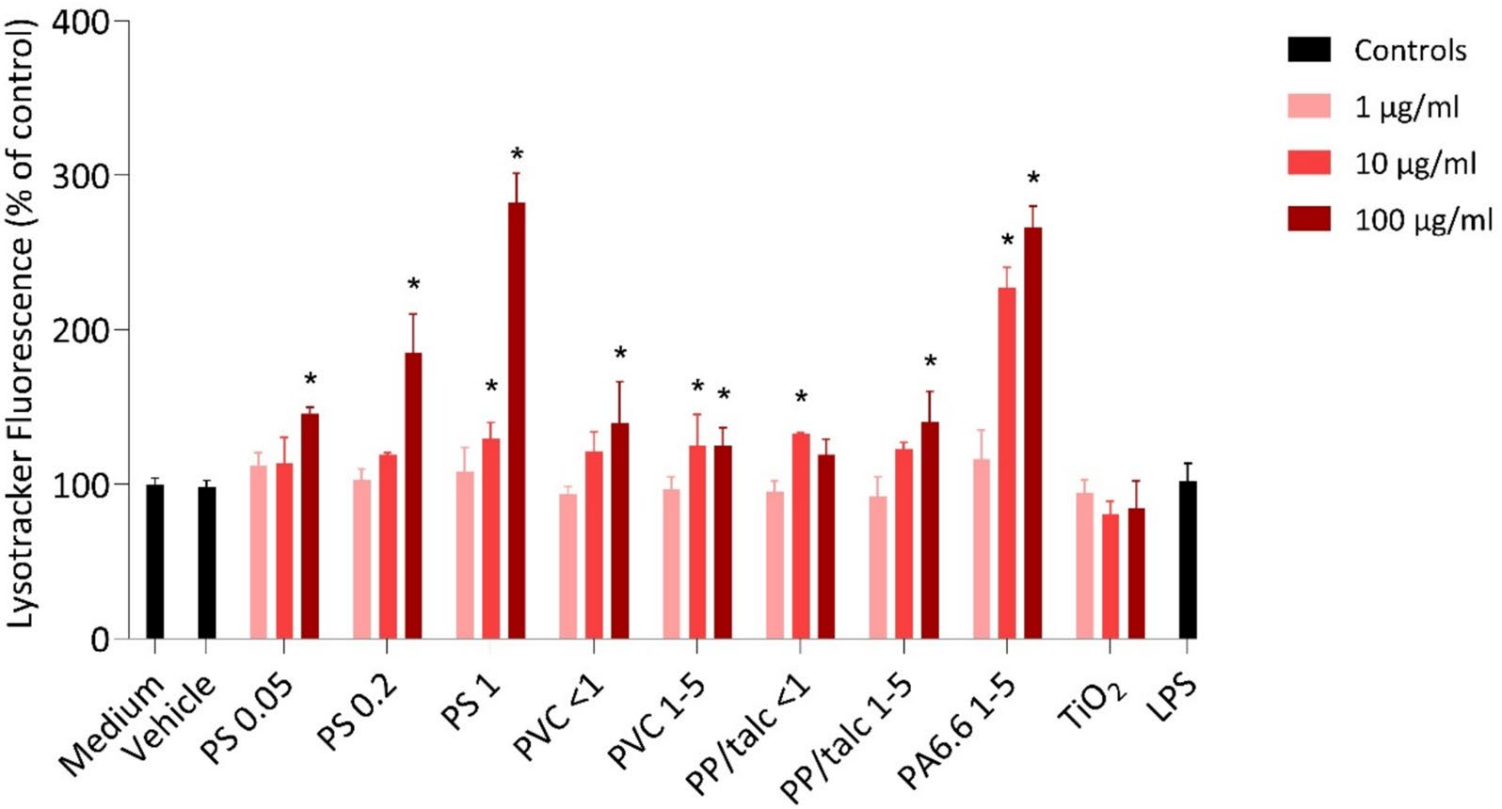
Increased Lysotracker Red fluorescence in THP-1 macrophages after 24 h of stimulation with varying concentrations (1, 10 and 100 µg/ml) of PS 0.05 µm, PS 0.2, PS 1 µm, PVC < 1 µm, PVC 1–5 µm, PP/talc < 1 µm, PP/talc 1–5 µm, PA6.6 1–5 µm, TiO_2_ and LPS (10 ng/ml). Data are presented as mean ± SD, *n* = 3. * *p* < 0.05 vs control

Exposure to all MNPs resulted in a dose-dependent increase in Lysotracker Red fluorescence, suggesting an increased volume of acidic compartments (Fig. 5). This data indicates that, after uptake, the MNPs interact with lysosomes. TiO_2_ and LPS did not show a significant effect on the Lysotracker fluorescence. Particularly noteworthy is the pronounced effect seen with the different sizes of PS particles and PA6.6 particles. The size-dependent increase observed with PS particles underscores the relationship between particle size and cellular response, with larger PS particles showing greater lysosomal activation compared to smaller ones. However, this size-dependent pattern is not clearly visible with the fragmented PP/talc and PVC, which might be due to the agglomeration of the particles.

### Effects of MNPs on cell viability

**Fig. 6:**
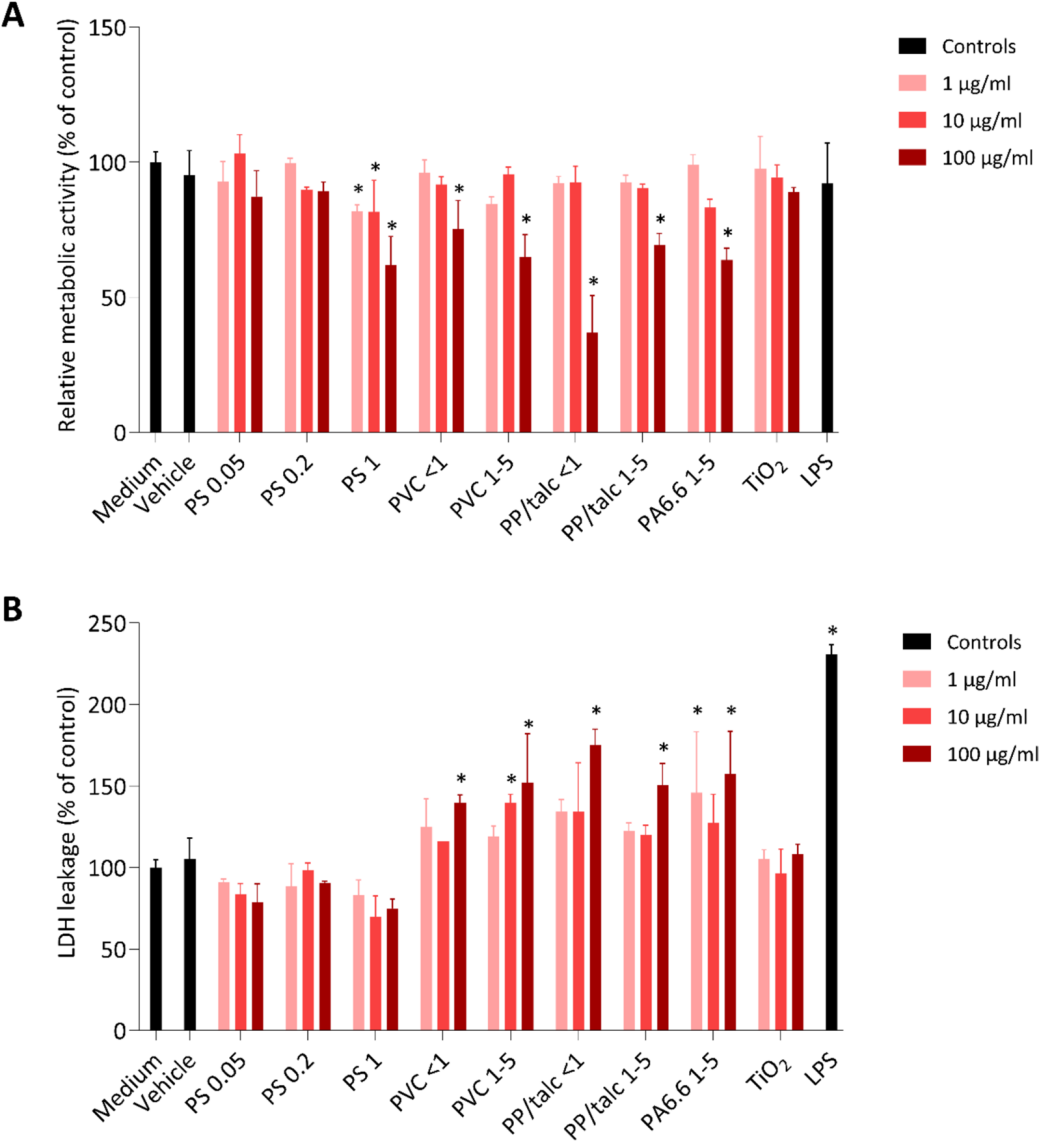
Cell viability of THP-1 cells treated with varying concentrations (1, 10 and 100 µg/ml) of PS 0.05 µm, PS 0.2, PS 1 µm, PVC < 1 µm, PVC 1–5 µm, PP/talc < 1 µm, PP/talc 1–5 µm, PA6.6 1–5 µm, TiO_2_ and LPS (10 ng/ml) as measured by relative metabolic activity (**A**) and LDH leakage (**B**). Data are presented as mean ± SD, *n* = 3. * *p* < 0.05 vs control

After 24 h exposure of THP1-Blue cells to varying concentrations (1, 10 and 100 µg/ml) of the different MNP types and sizes, we assessed cell cytotoxicity using two complementary assays. First, cellular metabolic activity was measured by AlamarBlue assay as an indication of mitochondrial condition. Relative metabolic activity was decreased for PS 1 µm, PVC < 1 µm, PVC 1–5 µm, PP/talc < 1 µm, PP/talc 1–5 µm and PA6.6 1–5 µm, particularly at 100 µg/ml. No effect was observed for PS 0.05 µm, PS 0.2 µm and TiO_2_ (Fig. 6A). Milled talc particles were tested as a non-MNP control and showed no significant decrease in cell viability. These findings suggest that mitochondrial activity is decreased by the fragmented MNPs in a dose-dependent manner.

To provide further insights into the cytotoxic potential of MNPs, the membrane integrity of macrophages was evaluated by analyzing LDH leakage. None of the PS particles showed a significant increase compared to the control, but all of the fragmented MNPs demonstrated significant LDH leakage at 100 µg/ml, indicating compromised cell membrane integrity (Fig. 6B). PVC 1–5 µm and PA6.6 1–5 µm also showed LDH leakage at lower concentrations, being 10 µg/ml and 1 µg/ml respectively. The results of the LDH leakage assay align with the AlamarBlue assay results, indicating that these fragmented MNPs adversely affect cell viability by both impairing mitochondrial function and compromising membrane integrity.

### NF-κB activation and inflammatory cytokine response after MNP exposure

**Fig. 7:**
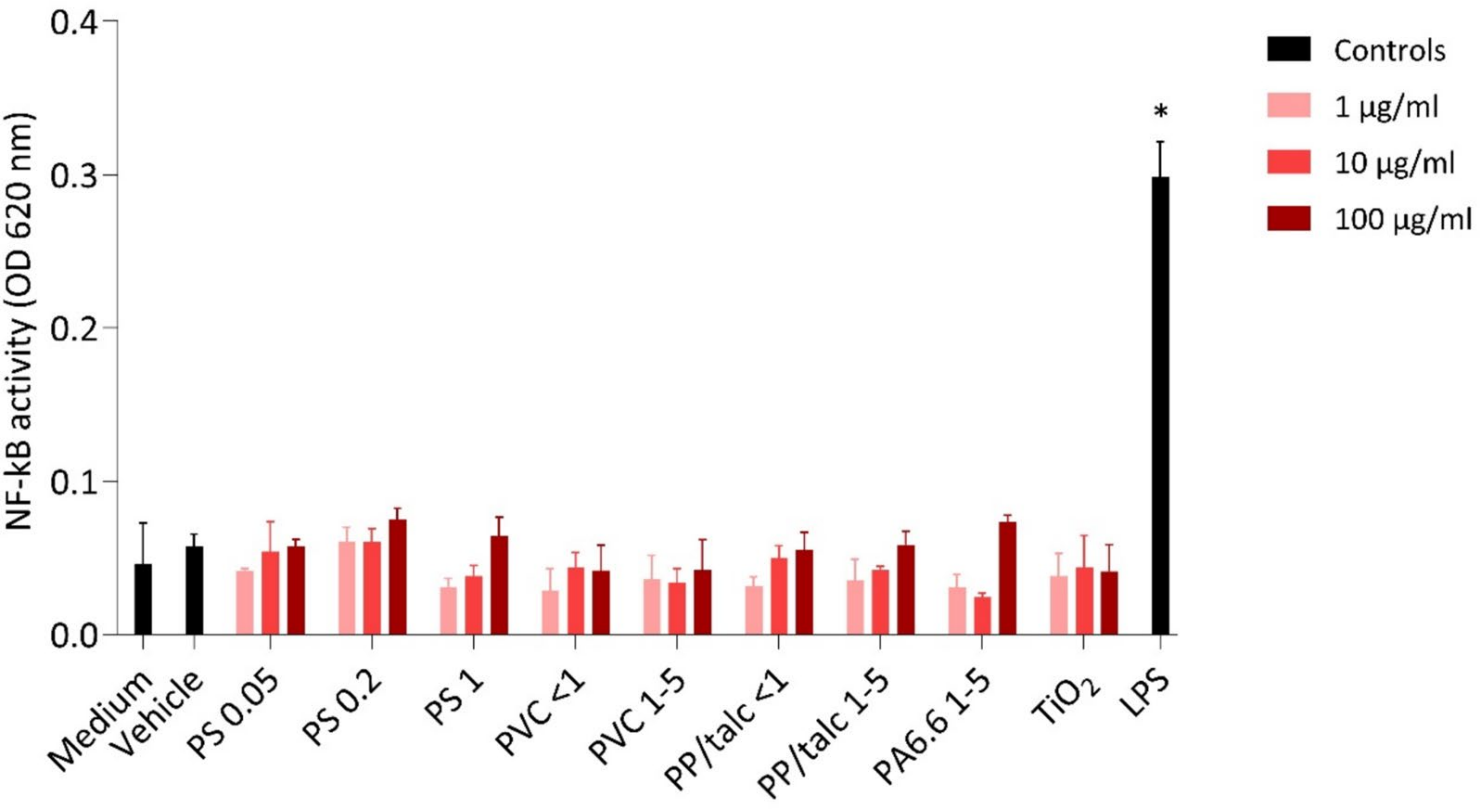
NF-κB activity in THP1-Blue cells exposed to 1, 10 and 100 µg/ml of PS 0.05 µm, PS 0.2, PS 1 µm, PVC < 1 µm, PVC 1–5 µm, PP/talc < 1 µm, PP/talc 1–5 µm, PA6.6 1–5 µm, TiO_2_ and LPS (10 ng/ml). The results are presented as mean ± SD, *n* = 3. * *p* < 0.05 vs control

To evaluate the potential pro-inflammatory effects of MNPs, NF-κB activation was measured using a SEAP reporter construct in THP1-Blue macrophages. Macrophages were exposed to various types of MNPs for 24 h to concentrations ranging from 1 to 100 µg/ml. None of the MNPs, regardless of size, concentration or polymer type, showed any significant effects on NF-κB activity compared to the medium and vehicle control. The positive control (LPS; 10 ng/ml) did show a significant increase, confirming THP1-Blue responsiveness (Fig. 7). In summary, these findings suggest that exposure to diverse MNPs does not influence NF-κB activity in macrophages.

To further examine the inflammatory pathways, levels of TNF-α, IL-6, and IL-1β were measured by ELISA assay in supernatant of THP-1 macrophages following the same 24 h exposure to the selected MNPs. Consistent with the NF-κB findings, none of the MNPs tested affected the release of cytokines (Figure S4).

## Discussion

This study provides new insights into the impact of mechanically fragmented MNPs derived from PVC, PP/talc, and PA6.6 on THP-1 macrophages. We show that exposure to these MNPs increases lysosomal activity, decreases mitochondrial activity and increases LDH leakage within 24 h of exposure. The lack of changes in NF-κB activity or cytokine production (IL-6, IL-1β, and TNFα) suggests that fragmented MNPs do not elicit a pro-inflammatory cytokine response under the tested concentrations. Although a direct endotoxin assay was not performed, the absence of significant NF-κB activation and cytokine production in our experiments indicates that any residual endotoxin in the particle suspensions is below the threshold needed to elicit an immune reaction.

Our uptake experiments revealed a time- and concentration-dependent uptake of all three sizes of PS particles. Confocal microscopy confirmed that the particles were located intracellularly, validating flow cytometry results and excluding only surface adherence*.* Our results regarding uptake of PS by phagocytotic cells align with previous studies, including our own [15, 17, 32–37] and support models suggesting that particle uptake depends on shape, size, rigidity, and surface roughness [38]. However, extrapolation of these findings to fragmented MNPs of different polymer types remains challenging due to the lack of reliable labelled materials that maintain environmental relevance, as well as due to the differences in physical behaviour such as agglomeration and sedimentation dynamics.

All tested MNPs, especially PS and PA6.6, showed a dose-dependent increase in lysosomal fluorescence, indicating transport to lysosomes for processing, which has been shown previously with 50 and 500 nm PS particles [39]. Similar to our results, it has been demonstrated that primary PS engage with lysosomes without affecting mitochondrial function [40], and that nanoparticles can induce lysosomal swelling, damage and apoptosis [41]. We observed a size-dependent increase in lysosomal fluorescence with the PS particles. It is well-established that smaller particles (< 0.05 μm) are passively taken up, whereas larger particles are actively taken up, leading to their accumulation in lysosomes [18, 19, 42]. This may explain why we observe greater lysosomal fluorescence with the larger PS particles. The comparatively lower increase seen with the fragmented particles, except for PA6.6, could suggest reduced uptake, potentially due to variations in dosimetry. The biological relevance of lysosomal activation, especially during chronic exposures or when co-stimulated with other environmental stressors that affect antigen processing, needs further investigation.

In this study, cytotoxicity was assessed by measuring relative metabolic activity and LDH release. Concentrations of 100 µg/ml were found to affect cell viability for all fragmented MNPs tested. PVC 1–5 µm and PA6.6 1–5 µm also showed LDH leakage at lower concentrations, being 10 µg/ml and 1 µg/ml respectively. These results demonstrate that fragmented MNPs can affect cell viability and membrane integrity in a concentration-dependent manner. In general, the effect of primary PS particles on macrophage cell viability or immune parameters has been reported only for very high concentrations, i.e. 250 µg/ml [33] or 500 µg/ml [43] and not at lower concentrations ranging from 0.01 to 200 µg/ml [15, 33, 44]. Moreover, previous studies show a greater hazard potential associated with fragmented MNPs [23, 45].

In our previous study, we found that environmentally weathered PS could activate dendritic cells [17]. Therefore, we hypothesized that secondary MNPs might similarly provoke immune activation in macrophages. Nevertheless, despite the cytotoxic effects, none of the tested MNPs activated the NF-κB signaling pathway or induced the release of key pro-inflammatory cytokines (IL-6, IL-1β, TNF-α), which aligns with previous research [23, 45]. The discrepancy with our initial hypothesis might suggest that mechanical fragmentation may differ from more complex weathering processes, such as oxidation and biofilm formation, that might alter surface chemistry and immunomodulatory capacity.

A key strength of this study is the use of MNPs with environmentally relevant features, including fragmented morphology, broad size distribution, and unmodified surfaces. However, these features also introduce complexity in interpreting results. Despite our best efforts, some common concerns and limitations in MNP research could not be circumvented. Given the absence of a consensus on dose metrics, we chose to expose the macrophages in a mass-based manner, since the detection in human matrices is also described in terms of mass concentrations [8]. However, this means that for the different sizes of MNPs in the same concentration, a different number of particles is present in the assays (Fig. 1C). The dosimetry modeling revealed variation in sedimentation rates and effective cell exposure, particularly for smaller or lower-density particles (Table 1). According to the DG model, the PVC and PA 6.6 fractions deposit almost all of their administered mass (≈ 95% and ≈ 87%, respectively), whereas PP/talc delivers only ≈ 45%. Measurable responses of PP/talc appear only at the highest tested dose of 100 µg/ml, i.e. ≈ 45 µg/ml delivered. Still, these responses occur at considerably higher doses than the levels at which PVC and PA 6.6 elicit their effects (≈ 9.5 µg/ml and ≈ 0.9 µg/ml, respectively). These comparisons should nevertheless be interpreted with care. The DG model treats particles and agglomerates as equivalent spheres. Although it uses the measured hydrodynamic diameter and effective agglomerate density to approximate porosity and protein corona, it still ignores the extra drag and orientation-dependent settling behaviour of non-spherical shapes. This suggests that the current dosimetry tools, designed for homogenous nanomaterials, still need improvement to accurately estimate the delivered dose of heterogeneous and complex MNPs.

Particle characterization further revealed that the smaller fragmented particles agglomerated in the medium. This was the case for PP/talc and PVC but not for PA6.6 and primary PS. These findings indicate that the effective particle size in the exposure medium can differ from that in the stock solution, potentially affecting the distribution and interaction of the particles with biological systems. Without accounting for these changes, comparisons across particle types or between studies may be misleading, potentially obscuring material-specific hazards. This underscores the urgent need for more straightforward approaches to accurately assess the hazards and risks associated with diverse MNPs. Vogel et al. proposed a practical framework that offers step-by-step guidance to address the challenges in the risk assessment of MNPs [46].

We differentiated THP-1 cells with 5 ng/ml PMA to produce an M0-like phenotype, providing a robust baseline for evaluating MNP-induced cytotoxicity and immunotoxicity. Although this approach serves as a solid starting point, future work could incorporate polarization-specific primary macrophages to capture the full spectrum of macrophage responses. In addition, long-term exposures and co-stimulation with other toxins may also reveal delayed or synergistic inflammatory responses not captured in short-term assays.

In conclusion, our findings highlight the importance of including fragmented MNPs in hazard assessment. While particles from PP, PVC, and PA6.6 induced measurable cytotoxicity, they did not elicit pro-inflammatory responses. The absence of significant pro-inflammatory effects suggests that the observed cytotoxicity may not be directly linked to immune activation. Yet, the cytotoxicity observed from fragmented MNPs could still cause an inflammatory response, such as neutrophil recruitment to sites of cell damage, a hypothesis that warrants further research. To fully understand the health risks posed by MNPs, it is crucial to further explore their complex toxicological profiles, particularly under environmentally relevant conditions.

## Supplementary Information

Supplementary Material 1. Figure S1. Dot plots and histograms showing differences in morphology and surface marker expression between undifferentiated and differentiated THP1 cells. Cells were initially gated using Forward Scatter Area (FSC-A) vs Side Scatter Area (SSC-A) (left hand plots), and then examined for CD14 surface marker expression. Figure S2. Number-based particle size distributions of samples in DMEM/F12 + 10% FBS, derived by Static Light Scattering (Mastersizer 3000). Three measurements are shown per sample. Figure S3. Confocal microscopy images of THP-1 macrophages exposed for 24 h to medium, 0.05, 0.2 and 1 µm YG fluorescent PS particles (10 µg/ml). Particles are shown in green, nuclei in cyan and actin in magenta. Figure S4. Cytokine release (TNF-α, IL-6, IL-1β) in THP1 cells exposed to varying concentrations (1, 10 and 100 µg/ml) of PS 0.05 µm, PS 1 µm, PVC < 1 µm, PVC 1-5 µm, PP/talc <1 µm, PP/talc 1-5 µm, PA6.6 1-5 µm, TiO_2_ and LPS (10 ng/ml). The results are presented as mean ± SD, n=3. * p < 0.05 vs control.

## Data Availability

Data are available on the eNanoMapper database, a public database hosting nanomaterials characterization data and biological and toxicological information. Datasets used and/or analysed during the current study are available from the corresponding author on reasonable request.

## Abbreviations

ELISA: Enzyme-Linked Immunosorbent Assay
FI: Fluorescence Intensity
IL: Interleukin
LDH: Lactate Dehydrogenase
LPS: Lipopolysaccharide
MNP: Micro- and Nanoplastic
PA6.6: Polyamide 6.6
PE: Polypropylene
PET: Polyethylene terephthalate
PMA: Phorbol 12-myristate 13-acetate
PP: Polypropylene
PS: Polystyrene
PVC: Polyvinyl chloride
RI: Refractive Index
SEAP: Secreted Embryonic Alkaline Phosphatase
SEM: Scanning Electron Microscopy
SLS: Static Light Scattering
THP-1: Human leukemia monocytic cell line
TiO₂: Titanium dioxide
TNF-α: Tumor Necrosis Factor-alpha
