# Supplementary material for: Top-down generated micro- and nanoplastics reduce macrophage viability without eliciting a pro-inflammatory response
Source: Microplast nanoplast. 2025 Aug 1;5(1):32. doi: 10.1186/s43591-025-00138-5 (PMC12316794; doi:10.1186/s43591-025-00138-5)

Supplementary Figures

Figure S1. Dot plots and histograms showing differences in morphology and surface marker expression between undifferentiated and differentiated THP1 cells. Cells were initially gated using Forward Scatter Area (FSC-A) vs Side Scatter Area (SSC-A) (left hand plots), and then examined for CD14 surface marker expression.

Figure S2. Number-based particle size distributions of samples in DMEM/F12 + 10% FBS, derived by Static Light Scattering (Mastersizer 3000). Three measurements are shown per sample.

Figure S3. Confocal microscopy images of THP-1 macrophages exposed for 24 h to medium, 0.05, 0.2 and 1 µm YG fluorescent PS particles (10 µg/ml). Particles are shown in green, nuclei in cyan and actin in magenta.

Figure S4. Cytokine release (TNF-α, IL-6, IL-1β) in THP1 cells exposed to varying concentrations (1, 10 and 100 µg/ml) of PS 0.05 µm, PS 1 µm, PVC < 1 µm, PVC 1-5 µm, PP/talc <1 µm, PP/talc 1-5 µm, PA6.6 1-5 µm, TiO_2_ and LPS (10 ng/ml). The results are presented as mean ± SD, n=3. * p < 0.05 vs control.

Figure S1.


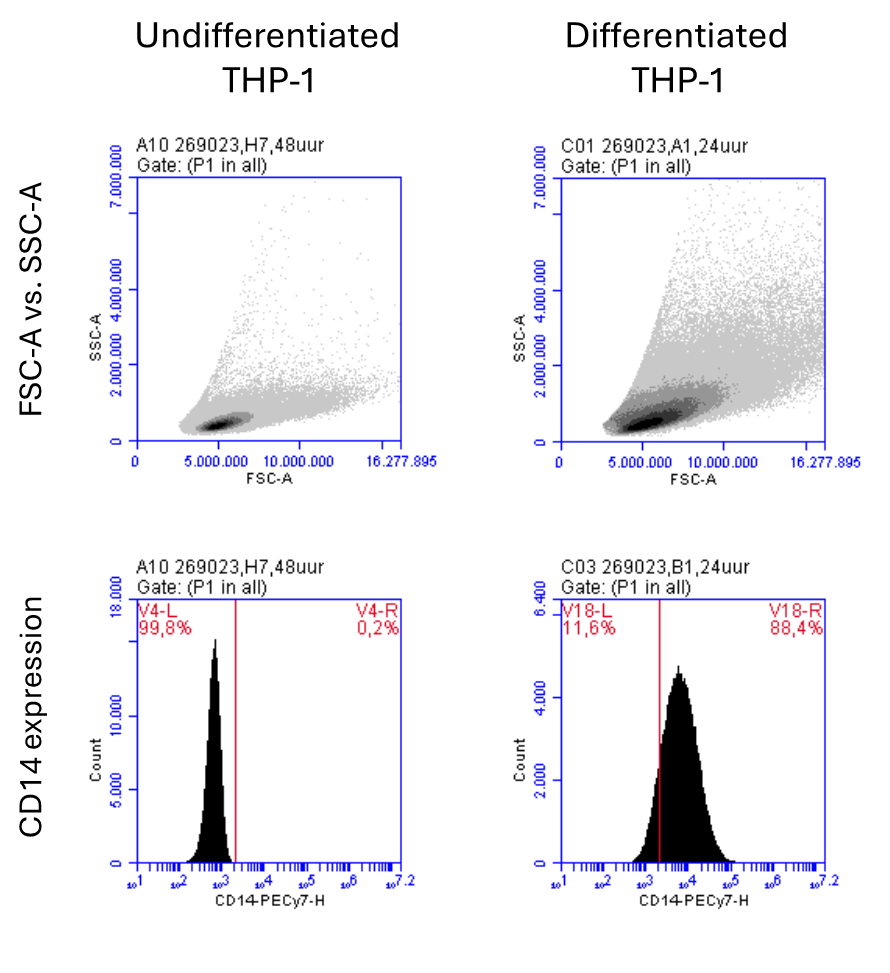


Figure S2.


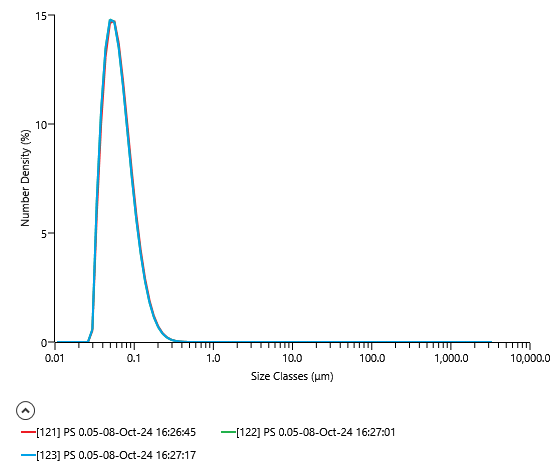

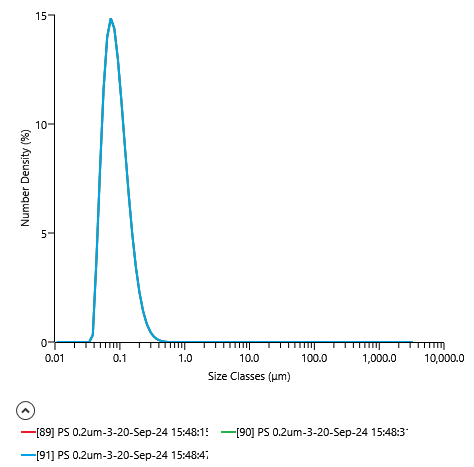

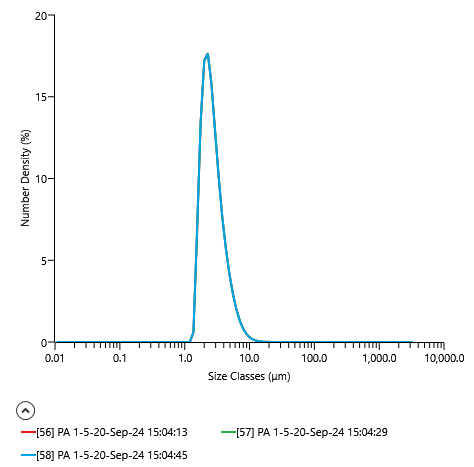

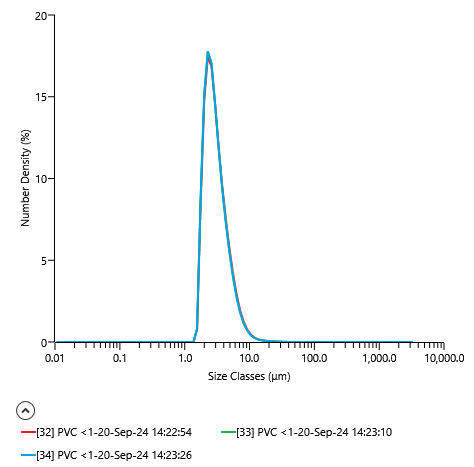

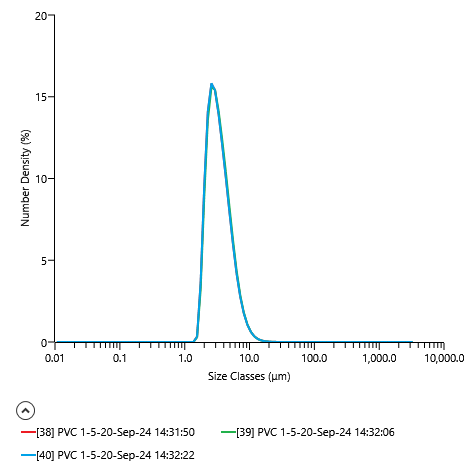

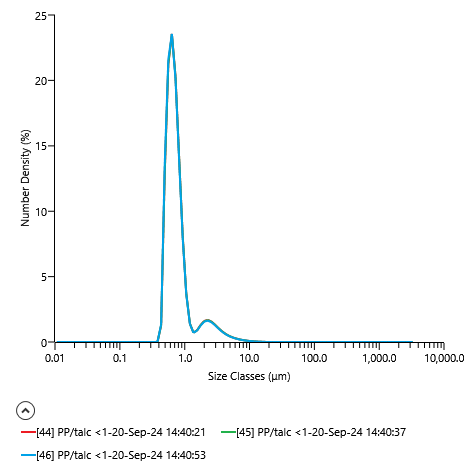


PS 0.05 µm

PS 1 µm

PS 0.2 µm

PVC <1 µm

PVC 1-5 µm

PP/talc <1 µm


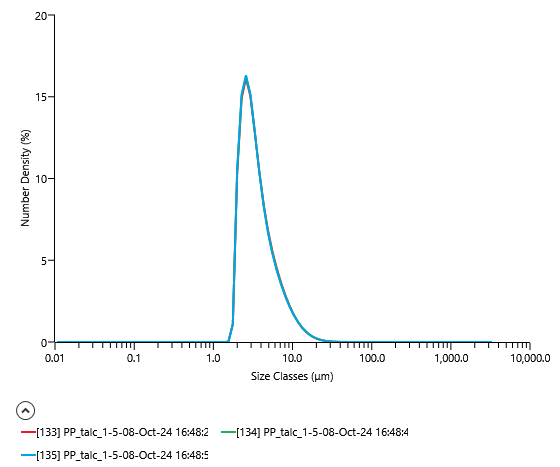

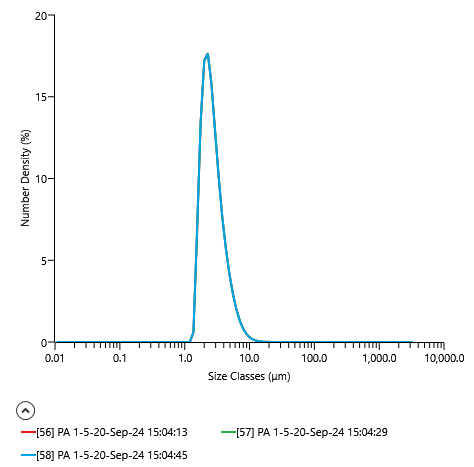


PP/talc 1-5 µm

PA6.6 1-5 µm

Figure S3.


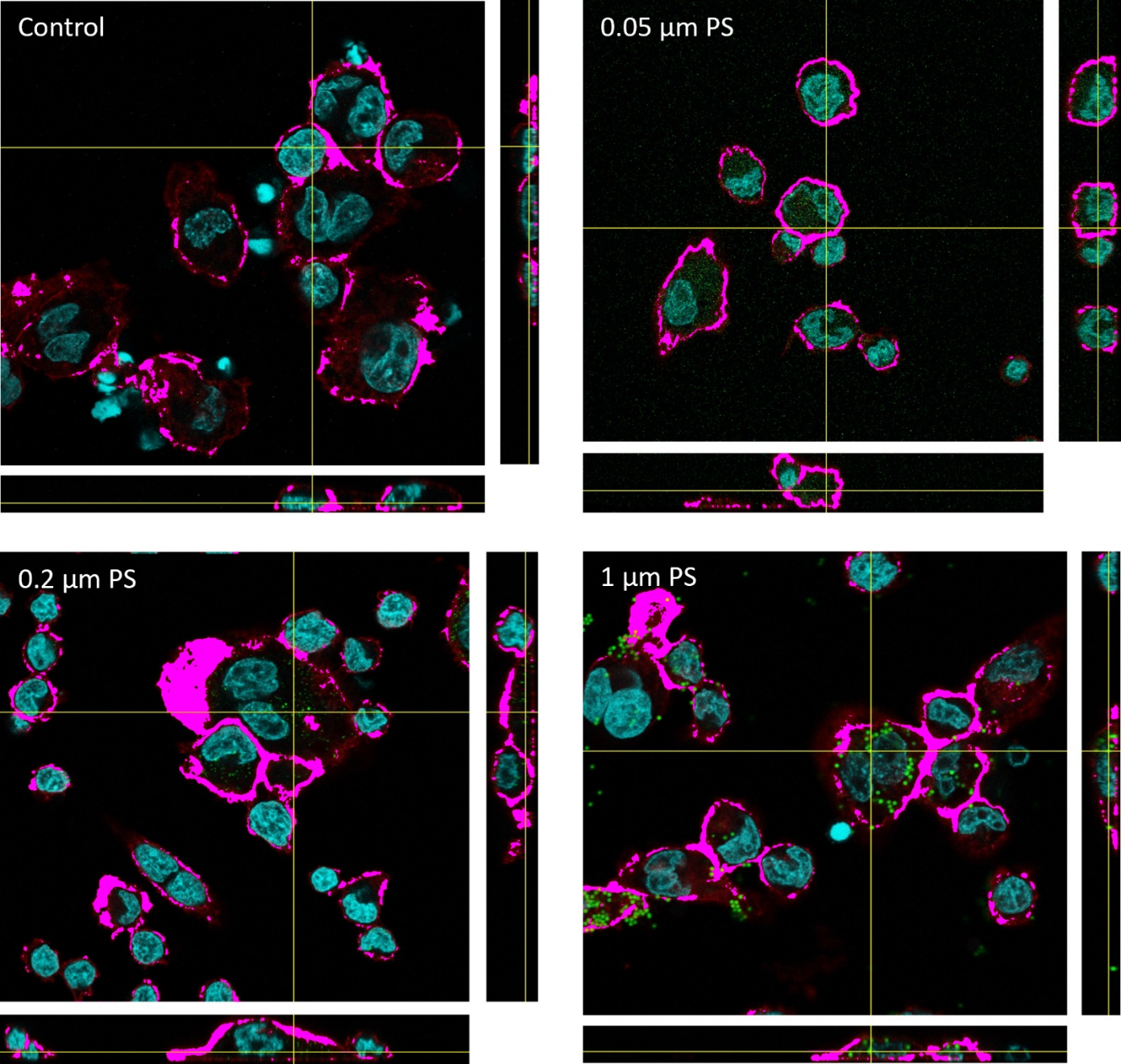


Figure S4.


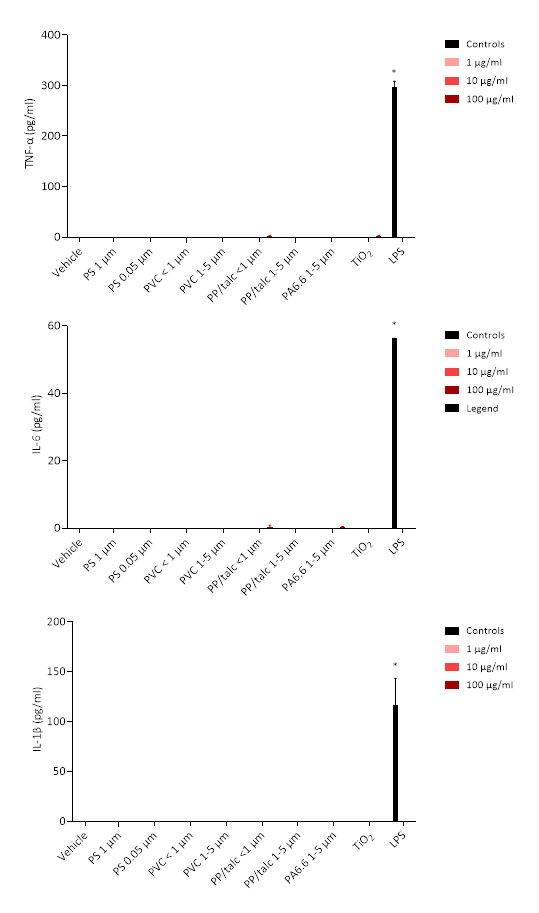

Supplement: Supplementary file 1 — Supplementary Material 1. Figure S1. Dot plots and histograms showing differences in morphology and surface marker expression between undifferentiated and differentiated THP1 cells. Cells were initially gated using Forward Scatter Area (FSC-A) vs Side Scatter Area (SSC-A) (left hand plots), and then examined for CD14 surface marker expression. Figure S2. Number-based particle size distributions of samples in DMEM/F12 + 10% FBS, derived by Static Light Scattering (Mastersizer 3000). Three measurements are shown per sample. Figure S3. Confocal microscopy images of THP-1 macrophages exposed for 24 h to medium, 0.05, 0.2 and 1 µm YG fluorescent PS particles (10 µg/ml). Particles are shown in green, nuclei in cyan and actin in magenta. Figure S4. Cytokine release (TNF-α, IL-6, IL-1β) in THP1 cells exposed to varying concentrations (1, 10 and 100 µg/ml) of PS 0.05 µm, PS 1 µm, PVC < 1 µm, PVC 1-5 µm, PP/talc <1 µm, PP/talc 1-5 µm, PA6.6 1-5 µm, TiO2 and LPS (10 ng/ml). The results are presented as mean ± SD, n=3. * p < 0.05 vs control. [file 43591_2025_138_MOESM1_ESM.docx]
